# Machine learning-based prediction model for postpartum stress urinary incontinence risk: a systematic review and meta-analysis

**DOI:** 10.3389/fmed.2026.1875678

**Published:** 2026-08-20

**Authors:** Xueling Zhong, Yutao Wang, Wenting Chai, Yanni Lin, Nengtong Zheng, Jinhua Huang

**Affiliations:** 1 School of Nursing, Fujian University of Traditional Chinese Medicine, Fuzhou, China; 2 The People’s Hospital Affiliated to Fujian University of Traditional Chinese Medicine, Fuzhou, China; 3 Graduate School, Fujian University of Traditional Chinese Medicine, Fuzhou, China

**Keywords:** machine learning, postpartum, prediction model, stress urinary incontinence, systematic review

## Abstract

**Background:**

Postpartum stress urinary incontinence (SUI) is a highly prevalent condition that imposes substantial physical, psychological, and economic burdens, underscoring the necessity of early identification of high-risk populations to improve clinical outcomes. However, existing machine learning (ML) prediction models yield inconsistent results, and their performance and reliability remain uncertain. This review aimed to synthesize the available evidence on ML-based prediction models for postpartum SUI.

**Objective:**

This study aimed to systematically evaluate the methodological quality, risk of bias, and predictive performance of ML-based prediction models for postpartum SUI, and to quantitatively synthesize their discrimination metrics.

**Methods:**

A systematic search of nine databases was conducted from inception to 23 March 2026. Studies developing and validating ML-based risk prediction models for postpartum SUI were included. Methodological quality and risk of bias were assessed using the PROBAST+AI tool, and reporting quality was evaluated with the TRIPOD+AI statement. A meta-analysis of the area under the receiver operating characteristic curve (AUC) was performed, employing robust variance estimation (RVE) to account for dependent effect sizes. The study was registered with PROSPERO (CRD420261369137).

**Results:**

Seven studies encompassing a total of 4,072 patients were included. All studies were rated as having a high risk of bias. The pooled AUC across 20 training models was 0.931 (95% CI: 0.880**–**0.962) and across 25 validation models was 0.890 (95% CI: 0.832**–**0.930). After correcting for dependent effect sizes applying RVE, the pooled AUCs were 0.915 (95% CI: 0.796**–**0.968) and 0.825 (95% CI: 0.637**–**0.927), respectively. Substantial heterogeneity was observed in both sets (*I*^2^ = 98.2 and 93.6%, respectively). Subgroup analyses revealed that models using only clinical features achieved the highest pooled AUC (0.974), whereas multimodal models integrating clinical features, pelvic floor ultrasound, and pelvic floor electromyography parameters showed lower AUC (0.821) but more stable performance (*I*^2^ = 50.3% vs. 95.2%). The most frequently included predictors were age, body mass index, parity, neonatal birth weight, and bladder neck descent.

**Conclusion:**

Current ML prediction models demonstrate acceptable discrimination for postpartum SUI, but they exhibit a high risk of bias, poor reporting standards, and a lack of external validation, rendering them not yet suitable for direct clinical application.

**Systematic review registration:**

https://www.crd.york.ac.uk/PROSPERO/view/CRD420261369137, CRD420261369137.

## 1 Introduction

Stress urinary incontinence (SUI) is a common condition among postpartum women and has become a global public health issue (1, 2). A systematic review encompassing 35,064 women reported a weighted average prevalence of urinary incontinence of 31.0% from 6 weeks to 1 year postpartum, with SUI accounting for up to 54% of cases (3). Another systematic review focusing on women in the first year postpartum also confirmed that the reported incidence of SUI ranged from 7.4% to 93.0%, similarly ranking first among all types of postpartum urinary incontinence (4). Beyond its adverse impact on women’s physical and mental health as well as quality of life (5, 6), it can also lead to social isolation (7–9) and increase the risk of postpartum depression (10, 11), while imposing a substantial economic burden. A large U.S. cohort study showed that patients with SUI/mixed urinary incontinence had 61% higher 2-year mean total medical costs compared with those without the condition (12). However, owing to insufficient disease awareness, shame, and financial pressure, the healthcare-seeking rate for postpartum SUI is generally low (13–16), and persistent symptoms further exacerbate the disease burden.

Early identification of high-risk individuals is key to reducing the incidence of postpartum SUI and improving clinical outcomes. Current screening methods such as questionnaires, pad tests, pelvic floor electromyography, pelvic floor ultrasound and urodynamic testing each have limitations: questionnaires are highly subjective; pad tests are susceptible to interference from factors such as fluid intake and activity level; pelvic floor electromyography cannot assess pelvic floor structures; although pelvic floor ultrasound provides anatomical information, its predictive performance for subclinical risk is limited when used alone (17, 18); and urodynamic testing, while the diagnostic gold standard, is difficult to employ as a routine screening tool because of its complexity and high cost. Therefore, integrating multidimensional heterogeneous data to develop an accurate, noninvasive early risk prediction tool holds substantial clinical value.

Prior researchers have conducted a systematic review of postpartum SUI risk prediction models built using conventional logistic regression, reporting model area under the receiver operating characteristic curve (AUC) values ranging from 0.602 to 0.888 (19). While logistic regression serves as a well-established statistical method, it is limited by its linear modeling assumptions, making it difficult to effectively capture complex nonlinear relationships and interaction effects among multiple variables, and its generalizability in external validation is often limited by insufficient sample size or suboptimal feature selection.

Machine learning (ML) is an important branch of artificial intelligence that constructs algorithms enabling computers to automatically learn patterns and regularities from data to make predictions or decisions without explicit programming. Unlike traditional statistical modeling, a core strength of ML is its ability to efficiently handle high-dimensional, multi-source data and automatically identify complex nonlinear relationships and deep interaction effects among variables. However, the boundary between machine learning and statistical (regression-based) prediction methods remains unclear, and the distinction is often cultural rather than methodological; for example, logistic regression is frequently claimed by both fields (20). Commonly used algorithms, such as decision trees, support vector machines, and random forests, have achieved notable success in risk prediction for various diseases (21–23). Researchers have begun to explore the application of these algorithms to postpartum SUI risk prediction, showing preliminary potential clinical value (24). However, these ML-based postpartum SUI prediction models exhibit considerable heterogeneity in study populations, predictor selection, algorithm strategies, sample size calculation, and performance validation methods, and their methodological quality and risk of bias have not yet been systematically assessed. Directly applying existing models to clinical decision-making may introduce bias due to potential methodological flaws, compromising their true predictive performance and generalizability. Currently, there is a lack of systematic evaluation and quantitative synthesis of ML prediction models for postpartum SUI.

Therefore, this study aims to conduct a systematic review of ML prediction model studies in the field of postpartum SUI using PROBAST+AI, a risk-of-bias assessment tool specifically designed for artificial intelligence-based prediction models. We comprehensively evaluate their methodological quality and risk of bias and conduct a meta-analysis of model performance metrics, thereby providing higher-quality evidence to support the early identification of high-risk populations and precise clinical interventions.

## 2 Materials and methods

This review was registered with the International Prospective Register of Systematic Reviews (PROSPERO) (CRD420261369137) and is reported in accordance with the Preferred Reporting Items for Systematic Reviews and Meta-Analyses (PRISMA) statement (Supplementary material S1).

### 2.1 Search strategy

A systematic search was conducted in PubMed, Web of Science, Embase, the Cochrane Library, Scopus, CNKI, Wanfang Data, VIP, and SinoMed from database inception to March 23, 2026, and the search has already captured the latest evidence before submission. The search strategy combined Medical Subject Headings and Emtree Terms with free-text keywords, including “stress urinary incontinence,” “machine learning” and “prediction model.” Reference lists of the included studies were also manually screened to identify any additional eligible studies. The full search strategy is provided in Supplementary material S2.

### 2.2 Inclusion criteria

1. The study population consists of postpartum adult women (≥18 years).
2. The study develops and validates a risk prediction model for postpartum stress urinary incontinence using machine learning algorithms.
3. The study design is a case–control study or a cohort study.
4. The outcome is the occurrence of postpartum stress urinary incontinence.

### 2.3 Exclusion criteria

1. The full text is unavailable, or key data are missing and cannot be obtained even after contacting the authors.
2. The article does not describe the model development process or methods.
3. Studies using only logistic regression without comparison to other ML approaches (25).
4. Studies published in languages other than English or Chinese.
5. Duplicate publication.

### 2.4 Literature screening

All retrieved records were exported from the databases and imported into the reference management software EndNote X9 to enhance the efficiency of literature screening. Duplicate records were identified and removed. Literature screening was performed independently by two reviewers. Initial screening was conducted by reviewing titles and abstracts, and full-text screening was completed by reviewing the full texts. Any disagreements were resolved through discussion, with adjudication by a third reviewer when necessary.

### 2.5 Data extraction

Data extraction was conducted in accordance with the Checklist for Critical Appraisal and Data Extraction for Systematic Reviews of Prediction Modeling Studies (CHARMS) framework (26). One reviewer independently extracted data from each included study, and a second reviewer verified the extracted data for accuracy and completeness. In cases where the full text was inaccessible or the published data were incomplete, we contacted the corresponding authors to request supplementary information. The extracted data encompassed the following domains: study characteristics, participant characteristics, predictor and outcome definitions, and model development and validation methodology, among others.

### 2.6 Assessment of study quality and risk of bias

Two reviewers independently evaluated the studies using the PROBAST+AI and TRIPOD+AI frameworks. A third reviewer adjudicated any ambiguous bias assessments.

The PROBAST+AI is an update and extension of the 2019 PROBAST tool, specifically designed for the systematic evaluation of clinical prediction model studies developed using traditional regression methods or artificial intelligence (27). The tool covers four key domains: participants and data sources, predictors, outcome, and analysis. It systematically evaluates methodological quality during model development (16 signaling questions), risk of bias during model evaluation (18 signaling questions), and model applicability. Each item is judged as “yes/probably yes/probably no/no/no information/not applicable,” and each domain is assigned an overall rating of “low risk,” “high risk,” or “unclear.” The model development section focuses on the assessment of methodological quality, whereas the model evaluation section focuses on the assessment of risk of bias. Compared with PROBAST, PROBAST+AI adds evaluation of issues specific to AI models such as data leakage, handling of class imbalance, algorithmic fairness, and study reproducibility, and is applicable to systematic reviews of both diagnostic and prognostic models.

TRIPOD+AI is a reporting guideline for clinical prediction models updated from TRIPOD 2015 by the Collins team in 2024, applicable to studies that develop or evaluate (validate) clinical prediction models using regression or machine learning methods (28). This guideline comprises 27 main items, covering title, abstract, introduction, methods, open science, patient and public involvement, results, and discussion. Compared with the previous version, TRIPOD+AI adds reporting requirements for data preprocessing, handling of class imbalance, model fairness analysis, code and data sharing, and model output probabilities and classification thresholds, thereby enhancing the transparency and reproducibility of machine learning prediction model studies.

### 2.7 Data synthesis and statistical analysis

All statistical analyses were performed using R software (version 4.4.3), with the “metafor” and “robumeta” packages employed to implement the core analyses. This study selected the AUC as the primary effect size to quantify model predictive performance, combined with 95% confidence intervals (CIs) to characterize the uncertainty of effect size estimates and thereby assess the stability of model performance.

Training models were developed and optimized using training datasets, whereas validation models were evaluated on independent internal or external validation datasets to assess generalizability and detect overfitting. Therefore, we conducted separate pooled analyses of AUC values and their 95% CIs for the training and validation sets. For included studies that did not report uncertainty metrics, the Hanley–McNeil method was used to estimate standard errors based on sample size and number of events (29). Given that some included studies developed multiple models using different algorithms based on the same dataset, this study built upon inverse variance weighting and further employed robust variance estimation to adjust for this non-independence (30–32), treating dataset and model as stratification factors to examine their potential interaction effects.

Heterogeneity was tested using the statistic, with values of 25, 50, and 75% representing low, moderate, and high heterogeneity, respectively. If *I*^2^ > 50%, a random-effects model was used for pooling; if *I*^2^ ≤ 50%, a fixed-effects model was adopted. Meta-regression and subgroup analyses were conducted to explore sources of heterogeneity, with meta-regression parameters estimated using the Knapp–Hartung adjustment method and maximum likelihood estimation (33). Sensitivity analysis was performed using the leave-one-out method to evaluate the robustness of the results. Egger’s test was used to assess publication bias; a *p*-value > 0.05 suggested a low probability of publication bias (34).

## 3 Results

### 3.1 Study selection

**Figure 1:**
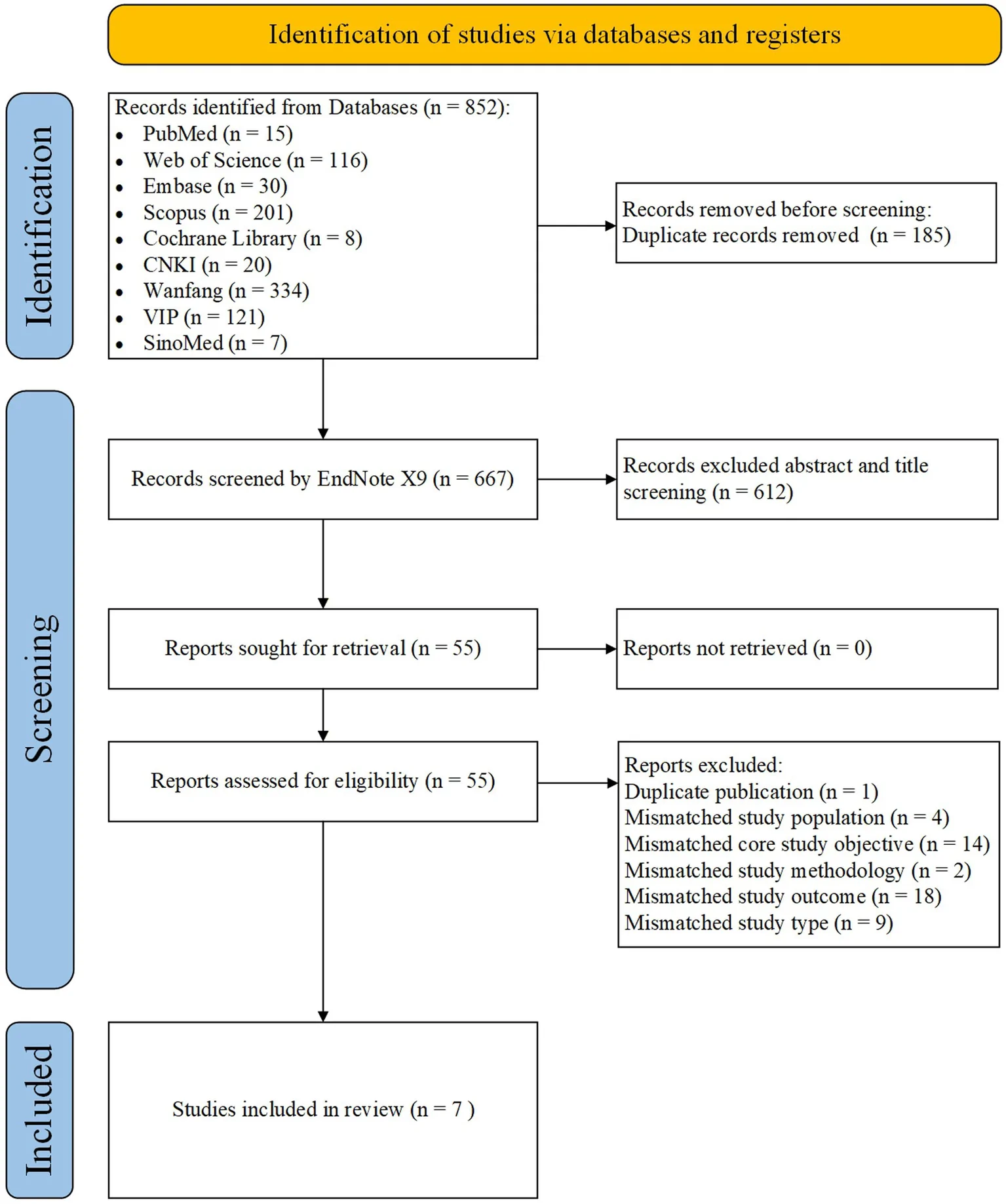
Search flow diagram.

A total of 852 records were identified through database searching, including the PubMed (*n* = 15), Web of Science (*n* = 116), Embase (*n* = 30), Scopus (*n* = 201), the Cochrane Library (*n* = 8), CNKI (*n* = 20), Wanfang (*n* = 334), VIP (*n* = 121), and SinoMed (*n* = 7). After removing 185 duplicate records, 667 records were screened by title and abstract, of which 612 were excluded. No reports were unretrievable. The remaining 55 full-text reports were assessed for eligibility. Of these, 48 reports were excluded for the following reasons: duplicate publication (*n* = 1), mismatched study population (*n* = 4), mismatched core study objective (*n* = 14), mismatched study methodology (*n* = 2), mismatched study outcome (*n* = 18), and mismatched study type (*n* = 9). Ultimately, seven studies met the inclusion criteria and were included in the meta-analysis (35–41). The PRISMA diagram is presented in Figure 1.

### 3.2 Characteristics of included studies

The seven included studies were all conducted in China and were all single-center studies. Upon data verification, no overlap in study populations or risk of data reuse was identified. The design of the included studies was predominantly retrospective; four were retrospective case–control studies (35, 37, 39, 41), two were retrospective cohort studies (36, 40), and one was a prospective cohort study (38). The sample sizes of the included studies ranged from 163 to 1,208 cases, and the prevalence of postpartum stress urinary incontinence ranged from 15.89% to 63.8%. The follow-up time points for SUI varied across studies, covering early postpartum periods (3 days, 42–60 days, 42–90 days, and 6–8 weeks) and late postpartum (12 months). All studies clearly reported the diagnostic criteria for SUI. Of these, two studies (37, 40) used the SUI diagnostic criteria established by the International Continence Society (ICS); two studies (35, 36) used the International Consultation on Incontinence Questionnaire-Urinary Incontinence Short Form (ICIQ-UI-SF) as the diagnostic tool; one study (38) employed the ICIQ-UI-SF combined with characteristic clinical symptoms of SUI for diagnosis; one study (41) adopted a comprehensive diagnosis combining the ICIQ-UI-SF, ICS criteria, and pelvic floor ultrasound; and one study (39) diagnosed SUI based on characteristic symptoms combined with positive results on the stress test and the Bonney test. Details are provided in Table 1.

**Table 1:** Basic characteristics of the included studies.

Study	Year	Country	Data source and study period	Study design	Study population	Incidence	Follow up duration	Primary outcome	Diagnostic criteria
Zhai et al. (35)	2020	China	Single-center NR 2014.9–2018.12	Retrospective case–control study	Postpartum women	204/707 (28.9%)	Within 3 days postpartum	PPSUI	ICIQ-UI-SF
Jiao et al. (36)	2022	China	Single-center Postpartum Rehabilitation Clinic of a tertiary A-level hospital in Shandong Province 2020.7–2021.1	Retrospective cohort study	Postpartum women	104/505 (20.6%)	6–8 weeks postpartum	PPSUI	ICIQ-UI-SF
Xu et al. (37)	2023	China	Single-center Pelvic Floor Clinic, Department of Obstetrics and Gynecology, Affiliated Hospital of Nantong University 2021.3–2022.11	Retrospective case–control study	Postpartum women	104/163 (63.8%)	NR	PPSUI	ICS criteria
Wang et al. (38)	2025	China	Single-center Postpartum Rehabilitation Clinic, Affiliated Hospital of Binzhou Medical University 2023.10–2024.5	Prospective cohort study	Postpartum women	406/1208 (33.6%)	Within 12 months postpartum	PPSUI	1. ICIQ-UI-SF; 2. Characteristic clinical symptoms of SUI
Shi et al. (39)	2025	China	Single-center Department of Urology and Department of Obstetrics and Gynecology, First Affiliated Hospital of Shihezi University 2019.10–2023.10	Retrospective case–control study	Postpartum women	352/611 (57.6%)	42–60 days postpartum	PPSUI	1. Characteristic clinical symptoms of SUI; 2. Positive stress test and Bonney test
Yang et al. (40)	2025	China	Single-center Pelvic Floor Center, Women and Children’s Hospital Affiliated to Ningbo University 2022.10–2023.6	Retrospective cohort study	Postpartum women	82/516 (15.9%)	42–90 days postpartum	PPSUI	ICS criteria
Wang et al. (41)	2025	China	Single-center Yinchuan Maternal and Child Health Hospital 2022.1–2023.12	Retrospective case–control study	Postpartum women	114/362 (31.5%)	Within 12 months postpartum	PPSUI	1. ICS criteria; 2. ICIQ-UI-SF; 3.pelvic floor ultrasonography

NR, not reported; PPSUI, postpartum stress urinary incontinence; ICIQ-UI-SF, international consultation on incontinence questionnaire-urinary incontinence short form; ICS, international continence society.

### 3.3 Characteristics of model development and validation

The seven included studies developed a total of 25 risk prediction models involving seven machine learning algorithms. Among these, decision trees were the most widely used. The sample size of the development sets ranged from 115 to 966 cases, and that of the validation sets ranged from 48 to 242 cases. Only two studies met the requirement of events per variable (EPV) ≥ 10 (38, 39); the remaining five studies had EPV below this threshold, suggesting a high risk of overfitting in most models. Regarding data preprocessing, four studies reported methods for handling missing data, with two using multiple imputation (38, 40). One study applied multiple imputation for variables with a missing proportion <10%, while also encoding categorical variables and standardizing continuous variables (41). Another study adopted a stratified strategy: variables with a missing rate >10% were directly deleted; for variables with a missing rate ≤10%, continuous variables with a normal distribution were imputed by the mean, those with a non-normal distribution by the median, and categorical variables by random imputation (39). For class imbalance, only two studies used the SMOTE oversampling technique (38, 41); the other five did not report any relevant methods. All studies used random splitting for internal validation. Among them, one study combined bootstrap resampling with 10-fold cross-validation (38); two studies used 5-fold cross-validation (39, 41); one study used 10-fold cross-validation (40); one study used cross-validation without specifying the number of folds (35); and two studies used random splitting only (36). Calibration assessment was severely lacking: only one study reported the calibration curve and Brier Score (41), while the remaining six studies did not perform any form of model calibration assessment. Details are provided in Table 2.

**Table 2:** Methodological and model characteristics of the included studies.

Study	Sample size (MD/IV)	EPV	Predictor selection method	Final predictors	Missing data handling	Class imbalance handling	Validation method	Calibration method	Model type	AUC	
										Training	Validation
Zhai et al. (35)	490/217	8	1. Univariate analysis; 2. Decision tree (stepwise entry method)	Pre-pregnancy BMI, Striae gravidarum, Constipation, Intensity of pelvic floor muscle training during pregnancy, Weekly fruit intake, Family history of urinary incontinence, History of abortion	NR	NR	Random split-sampling, cross-validation	NR	DT	0.602	0.767
Jiao et al. (36)	360/145	5.6	Univariate analysis	Parity, Postpartum BMI, Mode of delivery, Urinary incontinence during pregnancy, Endurance contraction EMG value	NR	NR	Random split-sampling	NR	DT	0.974	0.842
									LR	0.721	0.689
Xu et al. (37)	115/48	8.5	Lasso regression combined with ten-fold cross-validation	Maximum strength of fast-twitch (type II) muscle fibers, BND, Rate of urethral funneling, Retrovesical angle at maximal Valsalva maneuver	NR	NR	Random split-sampling	NR	ANN	0.870	0.857
									DT	0.856	0.746
									LR	0.815	0.876
									SVM	0.843	0.763
Wang et al. (38)	966/242	11	1. Univariate analysis; 2. Random forest feature importance ranking; the top ten variables were retained	Age at first delivery, Age at delivery, Pre-delivery BMI, Pre-pregnancy BMI, Parity, Neonatal birth weight, Gestational age, Gestational weight gain, Labor analgesia, Pre-delivery uterine height	Multiple imputation	SMOTE	Random split-sampling, bootstrap resampling, ten-fold cross-validation	NR	RF	0.990	0.995
									LR	0.976	0.978
									DT	0.935	0.948
									SVM	0.986	0.989
									XGB	0.988	0.993
Shi et al. (39)	428/183	11.7	Univariate analysis and Lasso regression	Age, BMI, Gravidity, Episiotomy, Maximum fast-twitch (type II) muscle strength, BND, URA, Constipation, History of urinary incontinence	For variables with more than 10% missingness: excluded. For variables with 10% or less missingness: mean imputation was used for normally distributed continuous variables, median imputation for non-normally distributed continuous variables, and random imputation for categorical variables	NR	Random split-sampling, five-fold cross-validation	NR	RF	0.944	0.906
									DT	0.815	0.750
									KNN	0.900	0.881
									SVM	0.925	0.878
Yang et al. (40)	361/155	2.8	1. Univariate analysis; 2. Random forest feature importance ranking; the top ten variables were retained	Age, BMI, Parity, Maximum neonatal birth weight, Perineal injury status, Endurance contraction phase, Tonic contraction phase, Pre-rest phase, Post-rest phase, Fast-twitch contraction phase	Multiple imputation	NR	Random split-sampling, ten-fold cross-validation	NR	SVM	0.896	0.779
									KNN	0.843	0.789
									XGB	0.999	0.772
									DT	0.734	0.701
									RF	1	0.837
Wang et al. (41)	253/109	3.4	Permutation importance for feature evaluation	Parity, Mode of delivery, Neonatal birth weight, Gestational weight gain, Gestational diabetes mellitus, BND, Levator ani muscle thickness, Aanal sphincter continuity, Levator hiatus area during Valsalva maneuver, Chronic cough	Multiple imputation	SMOTE	Random split-sampling, five-fold cross-validation	Calibration curve and Brier score	SVM	NR	0.924
									LR	NR	0.875
									RF	NR	0.900
									XGB	NR	0.931

Validation method: all studies used internal validation.

NR, not reported; BMI, body mass index; BND, bladder neck descent; URA, urethral rotation angle; DT, decision tree; SVM, support vector machine; RF, random forest; LR, logistic regression; XGB, eXtreme gradient boosting; KNN, K-nearest neighbor; ANN, artificial neural network.

### 3.4 Results of the PROBAST+AI assessment

For the model development studies, six were rated as having a high risk of bias (35–38, 40, 41), and one was rated as unclear (39). With regard to applicability, all seven studies were judged as having high applicability concerns. The main source of high risk of bias was the Analysis domain, as evidenced by failure to report the rationale for sample size estimation, lack of recalibration of predicted probabilities after class imbalance correction, unclear or missing descriptions of continuous variable preprocessing methods, and insufficient or ineffective overfitting control measures. Some studies showed near-perfect performance in the training set but a marked performance drop in the validation set, suggesting overfitting or potential data leakage. In addition, risks in the Predictors and Outcome domains stemmed primarily from the absence of blinded assessment in retrospective designs, inadequate reporting of data preprocessing details, and failure to clarify the independence of predictor or outcome assessment. The high concern for applicability mainly originated from the Participants domain, as all studies used a single-center design, which limits the external generalizability of the models.

**Figure 2:**
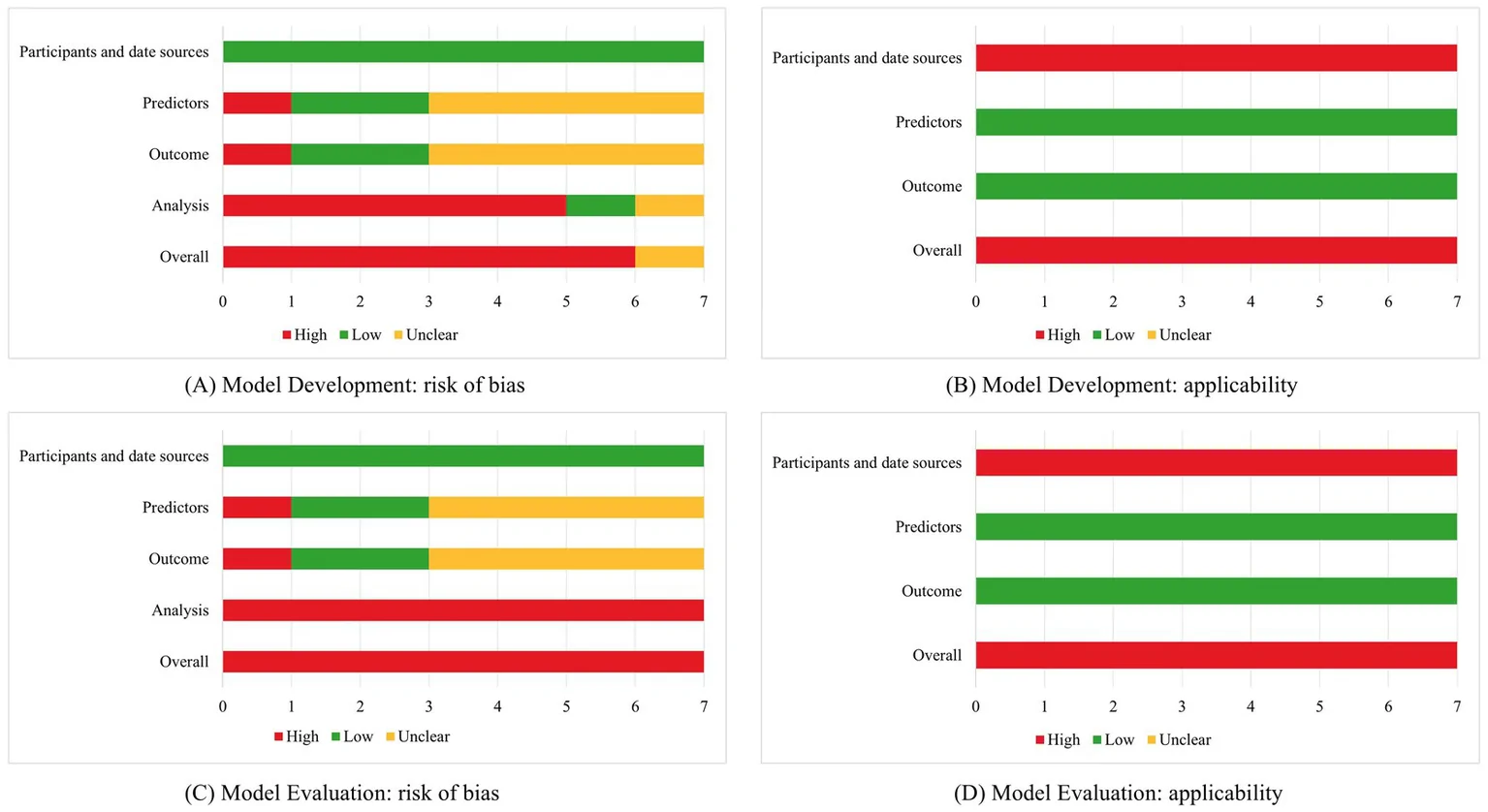
PROBAST+AI assessment results.

Among studies validating/evaluating prediction models, all seven were rated as having a high risk of bias, and all seven were judged as having high applicability concerns. High risk of bias was again concentrated in the analysis domain, with shared characteristics including: no reporting of model calibration (e.g., calibration curves or the Brier score) or clinical net benefit analyses, with evaluation largely confined to discrimination metrics (e.g., AUC); absence of justification for the adequacy of the evaluation sample size; failure to evaluate models on an independent test set that preserved the original class distribution after applying class imbalance correction, or failure to explicitly confirm that the test set was free from data leakage; and, in a small number of studies, a pronounced performance gap between the training and validation sets, further pointing to possible overfitting or data leakage. Risks in the predictor and outcome domains were primarily associated with the absence of blinding in retrospective designs and insufficient reporting of preprocessing procedures. With respect to applicability, all validations were based on single-center data and lacked any form of external validation, precluding an assessment of the models’ generalizability across different clinical settings. The results of the PROBAST+AI assessment are presented in Figure 2.

### 3.5 Results of the TRIPOD+AI assessment

The reporting quality of the seven included studies was evaluated using the TRIPOD+AI framework. The overall adherence rate was 58.9%, indicating widespread deficiencies in reporting transparency and completeness (Table 3). Among the sections, Title and Abstract achieved the highest adherence rates (both 100%), followed by the Introduction (75%), with relatively complete descriptions of the research background, clinical significance, and modeling objectives. However, none of the studies described health inequalities across sociodemographic groups (Item 3c), suggesting that equity considerations were overlooked at the study design stage.

**Table 3:** TRIPOD + AI checklist item.

Section/topic	Item	Development/Evaluation	Zhai et al. (35)	Jiao et al. (36)	Xu et al. (37)	Wang et al. (38)	Shi et al. (39)	Yang et al. (40)	Wang et al. (41)
Title									
Title	1	D;E	+	+	+	+	+	+	+
Abstract									
Abstract	2	D;E	+	+	+	+	+	+	+
Introduction									
Background	3a	D;E	+	+	+	+	+	+	+
	3b	D;E	+	+	+	+	+	+	+
	3c	D;E	−	−	−	−	−	−	−
Objectives	4	D;E	+	+	+	+	+	+	+
Methods									
Data	5a	D;E	+	+	+	+	+	+	+
	5b	D;E	+	+	+	+	+	+	+
Participants	6a	D;E	+	+	+	+	+	+	+
	6b	D;E	+	+	+	+	+	+	+
	6c	D;E	NA	NA	NA	NA	NA	NA	NA
Data preparation	7	D;E	+	−	+	+	+	+	+
Outcome	8a	D;E	+	+	+	+	+	+	+
	8b	D;E	−	−	−	+	−	−	−
	8c	D;E	−	−	−	−	−	−	−
Predictors	9a	D	+	+	+	+	+	−	−
	9b	D;E	+	+	+	+	+	+	+
	9c	D;E	−	−	−	+	−	−	−
Sample size	10	D;E	+	−	−	−	+	−	−
Missing data	11	D;E	+	+	−	+	+	+	+
Analytical methods	12a	D	+	+	+	+	+	+	+
	12b	D	−	+	−	+	+	−	+
	12c	D	+	+	+	+	+	+	+
	12d	D;E	−	−	−	−	−	−	−
	12e	D;E	+	+	+	+	+	+	+
	12f	E	NA	NA	NA	NA	NA	NA	NA
	12 g	E	+	+	+	−	+	−	−
Class imbalance	13	D;E	−	−	−	+	−	−	+
Fairness	14	D;E	−	−	−	−	−	−	−
Model output	15	D	+	+	+	−	+	−	−
Training versus evaluation	16	D;E	−	−	+	+	+	−	+
Ethical approval	17	D;E	−	+	−	+	+	+	+
Open science									
Funding	18a	D;E	+	−	−	+	−	+	+
Conflicts of interest	18b	D;E	−	−	−	+	−	+	−
Protocol	18c	D;E	−	−	−	−	−	−	−
Registration	18d	D;E	−	−	−	−	−	−	−
Data sharing	18e	D;E	−	−	−	+	−	−	−
Code sharing	18f	D;E	−	−	−	−	−	−	−
Patient and public involvement									
Patient and public involvement	19	D;E	−	−	−	−	−	−	−
Results									
Participants	20a	D;E	+	+	+	+	+	+	+
	20b	D;E	−	+	+	+	+	−	+
	20c	E	−	−	+	−	+	−	+
Model development	21	D;E	+	+	+	+	+	+	+
Model specification	22	D	+	+	+	−	+	−	−
Model performance	23a	D;E	+	+	+	+	+	+	+
	23b	D;E	−	−	−	−	+	−	−
Model updating	24	E	NA	NA	NA	NA	NA	NA	NA
Discussion									
Interpretation	25	D;E	+	+	+	+	+	+	+
Limitations	26	D;E	+	+	−	+	+	+	+
Usability of the model in the context of current care	27a	D	−	−	−	−	−	−	−
	27b	D	−	−	−	−	−	−	−
	27c	D;E	+	+	−	+	+	+	+

The adherence rate for the Methods section was 63.7%, with shortcomings concentrated in the following areas: severely inadequate justification of sample size (Item 10, 28.6%); complete absence of fairness assessment methods (Item 14, 0%); insufficient description of the pre-screening process for predictors (Item 9a, 71.4%); and a low reporting rate of class imbalance handling strategies (Item 13, 28.6%). These issues directly compromised the methodological rigor of model development and the credibility of the results. The adherence rate for the Results section was 69.4%. Although reporting rates for participant flow diagrams (Item 20a) and model performance metrics (Item 23a) reached 100%, the adherence rate for full model specification was only 57.1% (Item 22), and comparison of variable distributions between training and test sets (Item 20c) was reported in only 42.9% of studies, limiting readers’ ability to assess model applicability. The adherence rate for open science practices was 16.7%. Specifically, the adherence rates for sharing of study protocols (Item 18c), prospective registration (Item 18d), and sharing of analysis code (Item 18f) were all 0%; the adherence rate for data sharing statements (Item 18e) was 14.3%; and the adherence rate for conflict-of-interest statements (Item 18b) was 28.6%. These findings revealed serious deficiencies in computational reproducibility and research transparency. The adherence rate for the Patient and Public Involvement section (Item 19) was 0%. No study reported any stakeholder involvement in study design, conduct, reporting, or dissemination, further weakening the translational relevance of the research. The adherence rate for the Discussion section was 54.3%. Although 85.7% of studies discussed study limitations (Item 26), none addressed strategies for handling data quality issues during clinical deployment (Item 27a) or the level of expertise required by users (Item 27b), reflecting insufficient attention to the pathway from model development to clinical implementation.

**Figure 3:**
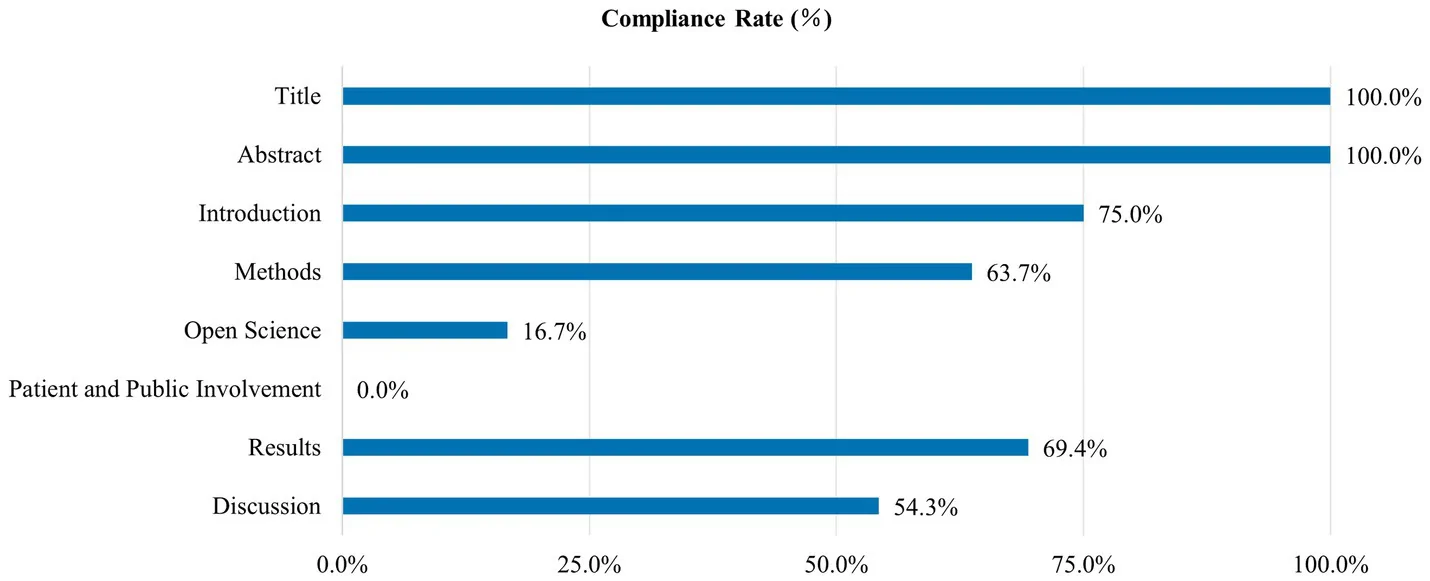
The compliance of TRIPOD+Al.

In summary, current studies on risk prediction models for postpartum stress urinary incontinence exhibited notable deficiencies in adherence to the TRIPOD+AI reporting guideline, with the weakest areas being open science practices, fairness assessment, sample size justification, and description of clinical implementation pathways. The systematic presence of these reporting gaps underscored the critical importance of rigorous adherence to the TRIPOD+AI statement for improving the scientific quality, reproducibility, and clinical translational value of research in this field. Detailed adherence rates are presented in Figure 3.

### 3.6 Predictors

Four studies adopted a two-stage hybrid strategy, of which two used univariate analysis followed by random forest feature importance ranking, retaining the top 10 variables (38, 40); one combined univariate analysis with a stepwise entry decision tree method (35); one integrated univariate analysis with Lasso regression (39). Two studies directly applied machine learning approaches, of which one implemented Lasso regression combined with 10-fold cross-validation (37); one used permutation importance for feature evaluation (41). One study used only univariate analysis for predictor selection (36). The number of predictors retained in the final models varied considerably across studies, ranging from 4 to 10. Despite methodological heterogeneity in selection strategies, there was notable consistency in the predictors commonly included across multiple studies. The five most frequent predictors were, in descending order: age, body mass index, parity, neonatal birth weight, and bladder neck descent. Among these, bladder neck mobility was the only ultrasound parameter commonly included in multiple studies, suggesting its unique value in predicting postpartum stress urinary incontinence. Given the high heterogeneity of predictor sets across studies and the unavailability of original data, this study did not perform a quantitative meta-analysis of individual predictors, but rather provided a descriptive synthesis.

### 3.7 Meta-analysis

During the data extraction and effect size synthesis process, some models were excluded owing to extreme asymmetry or boundary value issues in their reported confidence intervals. For example, the RF model constructed by Yang et al. (40) reported an AUC of 1 (95% CI: 1–1) in the training set, indicating a degenerate distribution with zero variance in the AUC estimate. Because this confidence interval precluded the calculation of a valid standard error, and including the model would compromise the variance estimation of the meta-analysis, it was removed from the pooled analysis. After this screening, the final meta-analysis included 20 models from 6 studies in the training set and 25 models from 7 studies in the validation set.

#### 3.7.1 Training models

**Figure 4:**
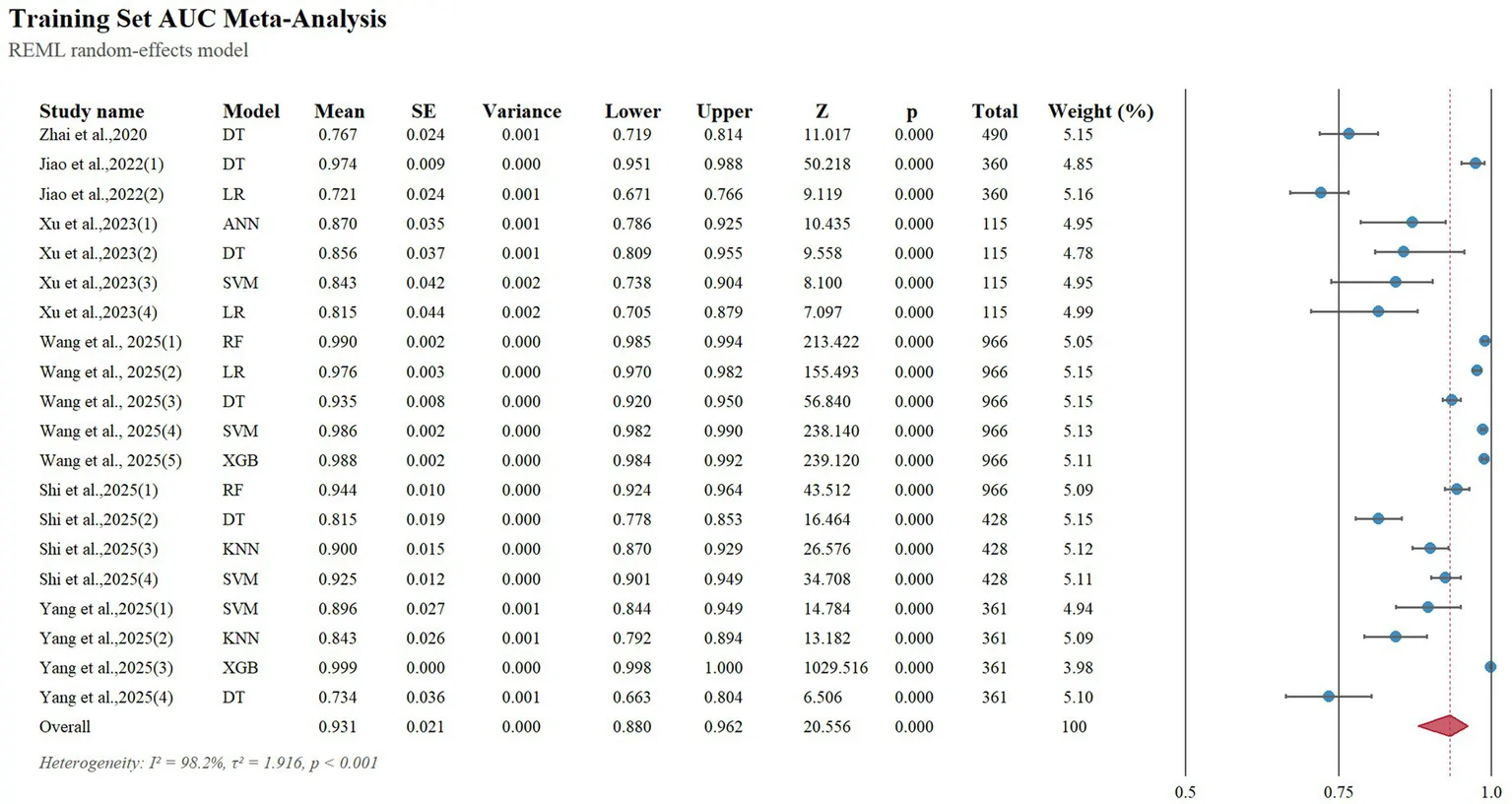
Forest plot of pooled AUC estimates from 20 training models under the random-effects meta-analysis.

A random-effects meta-analysis was performed on the 20 training models, yielding a pooled AUC of 0.931 (95% CI: 0.880–0.962) (Figure 4), suggesting that the included models demonstrated good discrimination on the development datasets. Leave-one-out sensitivity analysis showed that when individual models were sequentially removed, the pooled AUC ranged from 0.919 to 0.937, and the overall effect size was not substantially influenced by any single model, demonstrating the robustness of the results (Supplementary Figure S1). As shown in Supplementary Table S1A, variance decomposition based on dataset-model stratified effects found that AUC varied significantly across datasets (*σ* = 0.681), and there was a notable interaction effect between datasets and models (*σ* = 1.182). After correction using robust variance estimation, the pooled AUC was adjusted to 0.915 (95% CI: 0.796–0.968) (Supplementary Figure S2).

The *I*^2^ statistic for the training set was 98.2%, indicating high heterogeneity among the included models. Meta-regression revealed a significant positive association between sample size and pooled AUC (*β* = 0.003, *p* = 0.010) (Supplementary Table S2A), suggesting that sample size contributed to the observed heterogeneity. Leave-one-out analyses showed that the *I*^2^ statistic did not change substantially when individual models or entire studies were removed sequentially (Supplementary Table S3A). Furthermore, Egger’s test yielded an intercept of 0.203 (95% CI: −1.628, 3.415; *t* = 0.627; *p* = 0.370) (Supplementary Table S4). While the test did not detect statistically significant funnel plot asymmetry, with only seven independent studies, Egger’s test had limited statistical power to detect publication bias.

#### 3.7.2 Validation models

**Figure 5:**
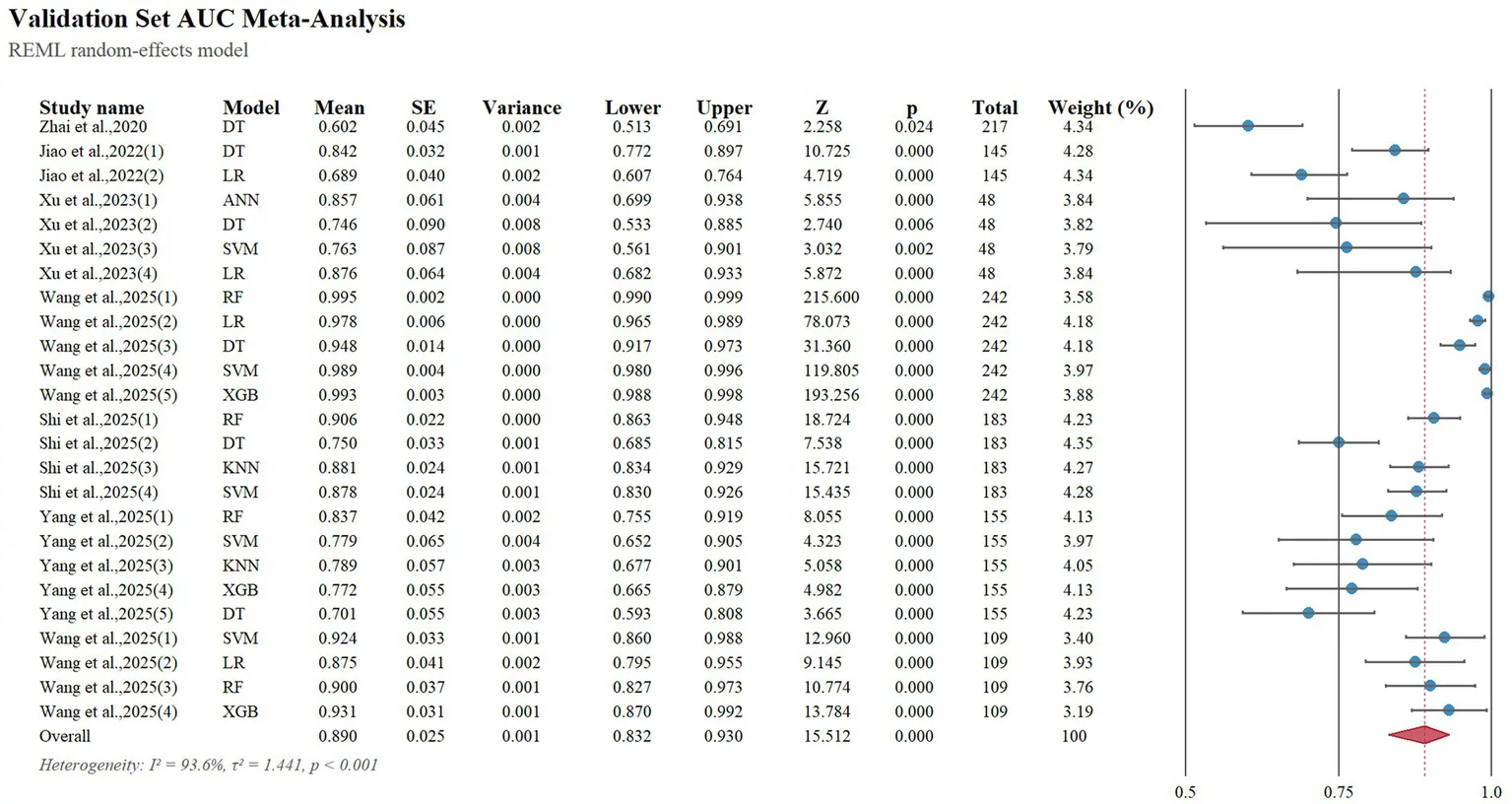
Forest plot of pooled AUC estimates from 25 validation models under the random-effects meta-analysis.

For the 25 validation models, the random-effects meta-analysis produced a pooled AUC of 0.890 (95% CI: 0.832–0.930) (Figure 5), suggesting that the included models demonstrated good discrimination on the validation set. Leave-one-out sensitivity analysis showed that when individual models were sequentially removed, the pooled AUC ranged from 0.877 to 0.897, indicating that the pooled estimate was robust (Supplementary Figure S3). In Supplementary Table S1B, the variance decomposition results showed that in the validation models, AUC varied across datasets (*σ* = 1.122) and there was evident interaction variation between datasets and models (*σ* = 0.144). After correction using robust variance estimation, the pooled AUC was adjusted to 0.825 (95% CI: 0.637–0.927).

The *I*^2^ statistic for the validation set was 93.6%, indicating high heterogeneity among the included models. Meta-regression showed significant positive associations of sample size (*β* = 0.010, *p* = 0.008) and publication year (*β* = 0.410, *p* = 0.024) with pooled AUC (Supplementary Table S2B), suggesting that both contributed to the observed heterogeneity. Leave-one-out analyses showed that the *I*^2^ statistic did not change substantially when individual models were sequentially removed; however, when all five models from Wang et al. (38) were excluded together, the *I*^2^ statistic decreased to 72.58% (Supplementary Table S3B). Furthermore, Egger’s test yielded an intercept of 0.325 (95% CI: −1.483, 2.133; *t* = 0.508; *p* = 0.640) (Supplementary Table S4). However, the limited number of included studies restricted the power of this test, and the possibility of publication bias could not be excluded.

#### 3.7.3 Subgroup analysis

Subgroup analyses of the training set (Supplementary Table S5) yielded the following results. When stratified by predictor type, models incorporating only clinical features achieved the highest pooled AUC of 0.970 (95% CI: 0.889–0.992). The multimodal combination of clinical features + PFEMG + PFU had a pooled AUC of 0.892 (95% CI: 0.818–0.938), whereas the clinical features + PFEMG subgroup yielded an extremely wide 95% CI of 0–1, indicating an unstable estimate. When stratified by the median sample size (394), the large-sample subgroup had a pooled AUC of 0.952 (95% CI: 0.892–0.979), while the small-sample subgroup had a pooled AUC of 0.892 (95% CI: 0.762–0.955). When stratified by model type, the pooled AUCs for various algorithms ranged from 0.870 to 0.996, with DT and SVM showing relatively stable performance.

Subgroup analyses of the validation set (Supplementary Table S6) yielded the following results. By predictor type, the models using only clinical features again had the highest pooled AUC of 0.974 (95% CI: 0.846–0.996); the clinical features + PFU subgroup had 0.900 (95% CI: 0.838–0.941); the multimodal combination of clinical features + PFEMG + PFU had a pooled AUC of 0.821 (95% CI: 0.771–0.862); and the clinical features + PFEMG subgroup had the lowest AUC of 0.772, with an extremely wide confidence interval (95% CI: 0.013–0.999). When stratified by the median sample size (155), the large-sample subgroup had a higher pooled AUC than the small-sample subgroup (0.910, 95% CI: 0.815–0.959 vs. 0.839, 95% CI: 0.768–0.891). By model type, the pooled AUCs ranged from 0.790 to 0.949, with DT, SVM, and RF showing relatively stable performance.

Whether in the training dataset or the validation dataset, the heterogeneity among models using only clinical features was extremely high (*I*^2^ = 98.4 and 95.2%, respectively). Although a positive trend between sample size and model performance was observed in both sets, the association did not reach statistical significance in either. DT was the most common algorithm; however, significant heterogeneity was observed for this subgroup in both sets (*I*^2^ = 93.2% in the training set and 88.6% in the validation set).

## 4 Discussion

This study is the first meta-analysis to quantitatively synthesize the predictive performance of ML-based risk prediction models for postpartum SUI and included 7 studies. Because most of the included models were derived from a limited number of datasets, the assumption of independent effect sizes in conventional meta-analysis is difficult to satisfy. The variance decomposition in this study indicated that models within the same study shared unmeasured design specific features; analyses that ignore such clustering effects effectively treat correlated observations as independent data, resulting in spuriously narrow confidence intervals. Robust variance estimation corrects for non-independence of effect sizes by adjusting standard errors and accounting for the hierarchical structure of the data, thereby providing more realistic estimates of the models’ generalizability and uncertainty across heterogeneous studies. This correction is especially crucial for meta-analyses of ML models, because it addresses pseudo-replication that arises when multiple models from a single study are treated as independent observations.

After applying the RVE correction, the pooled AUC decreased from 0.931 (95% CI: 0.880–0.962) to 0.915 (95% CI: 0.796–0.968) in the training set, and from 0.890 (95% CI: 0.832–0.930) to 0.825 (95% CI: 0.637–0.927) in the validation set. An AUC exceeding 0.80 is generally regarded as indicating acceptable discriminative performance (42). These results suggest that the ML models have acceptable average discrimination for postpartum SUI risk. However, AUC reflects only the model’s ability to distinguish between individuals with and without the outcome (43), it does not directly demonstrate clinical utility, which additionally requires evidence of adequate calibration, positive net benefit in decision curve analysis, and ultimately, improved patient outcomes in clinical implementation studies (44). The pooled AUCs of the ML models included in this meta-analysis were higher than those reported in previous systematic reviews of SUI prediction models developed using conventional statistical methods. For example, the review by Hou et al. (19) that included 15 maternal SUI prediction models with reported AUCs ranging from 0.602 to 0.888, and another systematic review that included 10 postpartum SUI prediction models with AUCs ranging from 0.680 to 0.850 (45), which to some extent reflects the potential advantage of ML algorithms in capturing complex non-linear relationships. However, the wider 95% CI for the validation set models in our study indicates suboptimal overall stability of the included models (30), which may be related to the relatively small validation sample size. Although the meta-analysis included 20 training models and 25 validation models, these were derived from only 7 independent study datasets. Consequently, the effective sample size at the study level is limited.

Studies with large sample sizes generally provide more comprehensive patient-level clinical data and support a more robust model training process, which often yields better goodness of fit during model development (46). Notably, in the small-sample subgroup, *I*^2^ decreased from 94.6% in the training set to 43.7% in the validation set. A possible reason for this substantial reduction in heterogeneity is that the validation set, as an independent external dataset, excluded the amplified random errors caused by insufficient sample size during training. Consequently, the performance of models from small-sample studies converged more in the validation stage than in the development stage. Nevertheless, the *I*^2^ for the small-sample subgroup remained above 40%, suggesting that insufficient sample size can still compromise the stability of model generalization.

With respect to predictor combinations, it is generally believed that integrating multidimensional data provides more comprehensive patient information and should theoretically improve predictive performance. However, our study showed that in the validation set, the pooled AUC of the multimodal combination of clinical features + PFU + PFEMG was markedly lower than that of the clinical features-only model; a similar trend was observed in the training set. This indicates that under the current data conditions, the addition of extra modal data did not yield the expected gain in average discrimination. This phenomenon may stem from an interplay of multiple factors. From the perspective of sample size relative to model complexity, incorporating high-dimensional imaging and electromyographic parameters substantially expands the model’s parameter space, while the EPV for most of the included studies was below 10, which places multimodal models at higher risk of overfitting in small cohorts. This risk is pervasive in the development of clinical prediction models, and it is even more pronounced for ML models, which have a greater capacity to fit noise compared with traditional regression. Therefore, strictly adhering to sample size recommendations and systematically evaluating performance variability are crucial for balancing model complexity and generalizability (47). Furthermore, PFU and PFEMG measurements are highly operator-dependent, and differences in device calibration and inter-institutional protocol heterogeneity may introduce additional noise in external validation (48–50). ML algorithms, given their capacity to model complex interactions, may be more susceptible to such technical variability than simpler models. Therefore, systematic assessment of data quality and standardization of procedures are key to further improving the performance of ML prediction models.

Although multimodal models did not show an advantage in average discrimination, their heterogeneity characteristics offer a complementary insight. In the validation set, the *I*^2^ for the multimodal model (clinical features + PFU + PFEMG) was 50.3%, markedly lower than that for the clinical features-only subgroup (95.2%); a similar pattern was observed in the training set. This finding suggests that incorporating objective imaging and electrophysiological markers helps calibrate measurement biases across studies, establishes a more standardized prediction framework with reduced performance variability, and partially offsets the random errors introduced by single-center studies or subjective indicators. Consequently, multimodal models tend to exhibit more stable performance across datasets. Therefore, when selecting prediction tools for clinical practice, one should consider not only the average discrimination but also the stability of performance across settings, which is particularly critical for multicenter generalization and primary care applications.

Variation in follow-up time points across the included studies is one source of clinical heterogeneity. These ranged from 3 days postpartum to 12 months postpartum. SUI occurring within days of delivery is often transient and may reflect acute neuromuscular or connective tissue injury that resolves with natural recovery, whereas SUI persisting at 12 months is more likely to represent chronic pelvic floor dysfunction (51). Pooling these outcomes under a single SUI definition may obscure important differences in underlying pathophysiology and clinical trajectory. However, due to the limited number of studies at each time point, a formal subgroup analysis by follow-up duration could not be performed, and this is one limitation. It is recommended that future prediction model studies adopt standardized, clinically meaningful follow-up windows to facilitate more granular evidence synthesis.

An additional source of heterogeneity that merits attention is the variation in outcome definitions across studies. The included studies employed different diagnostic criteria for postpartum SUI, ranging from questionnaires to clinical criteria, physical examination maneuvers, and combined ultrasound-based assessments. These instruments differ to some extent in measurement properties; such differences in case definition can lead to systematic variation in the composition of the SUI-positive groups across studies, which in turn affects the performance metrics of prediction models developed on these populations. This underscores the need for standardized, consensus-based outcome definitions in future postpartum SUI prediction research, ideally aligned with the ICS/IUGA joint terminology.

With respect to model type, decision tree was the most common algorithm; however, DT had relatively low AUCs across all algorithm categories and exhibited significant heterogeneity. Ensemble learning methods (RF and XGB) ranked among the highest pooled AUCs in both the training and validation sets. Nonetheless, the precision of their estimates was constrained by the small number of models per subgroup, resulting in very wide confidence intervals and insufficient evidence to confirm a general advantage. SVM demonstrated relatively robust performance in both datasets with a comparatively large number of included models, but it also exhibited high heterogeneity. In contrast, only a very limited number of KNN and ANN models were included in this analysis, resulting in insufficient statistical power and requiring extreme caution when interpreting their results. Therefore, based on the current data, it is premature to conclude that any single algorithm is definitively superior to the others.

Given the limited number of independent studies, both the meta-regression and Egger’s test results should be interpreted as exploratory only. Consequently, non-significant associations or tests should not be taken as evidence of absence, and significant associations may be disproportionately influenced by outlying data points rather than reflecting robust between-study relationships. Therefore, the exploratory associations identified, such as the positive relationship between sample size and pooled AUC, and the non-significant Egger’s test results, should be considered hypothesis-generating and require validation in future studies with larger study-level sample sizes.

A model with good discrimination but poor calibration can produce systematically biased risk estimates, leading to overtreatment or undertreatment (52). In the setting of postpartum SUI, an overestimating model may cause unnecessary anxiety and expose women to unwarranted interventions, whereas an underestimating model may deprive high-risk women of timely referral to pelvic floor rehabilitation. Among the seven studies included in this study, only one reported calibration assessment. While the pooled AUC of 0.82 indicates promising discriminative performance, a well-calibrated model with a lower AUC can be clinically preferable to a model with higher AUC but poor calibration (53). Therefore, future prediction model studies in postpartum SUI should consistently report calibration metrics alongside discrimination to facilitate meaningful appraisal of clinical utility.

Methodological quality and reporting quality were assessed using the PROBAST+AI and TRIPOD+AI frameworks, respectively. The PROBAST+AI results showed that six studies (85.7%) were rated as high risk of bias in the model development phase, and one (14.3%) had unclear quality risk; all seven studies were rated as high risk of bias in the model evaluation phase. The Analysis domain was the most concentrated source of risk of bias, with prominent issues including failure to report the rationale for sample size estimation, lack of recalibration of predicted probabilities after class imbalance correction, insufficient or ineffective overfitting control measures, and the systematic absence of calibration assessment and clinical net benefit analysis in the evaluation phase. These deficiencies were not isolated but interrelated, collectively undermining the reliability and clinical interpretability of model performance evaluation. Moreover, concern for applicability was equally serious: all studies were based on single-center data and lacked external validation, and the insufficient representativeness of the datasets severely constrained the generalizability of the models to different clinical settings.

With respect to reporting quality, the TRIPOD+AI assessment revealed an overall item compliance rate of only 58.9%. Although the compliance rates for Title and Abstract (100%) and Introduction (75.0%) were relatively acceptable, critical methodological details were substantially underreported. The low reporting rates for sample size justification (28.6%) and strategies for handling class imbalance (28.6%), together with the complete absence of fairness analysis methods, directly impeded a comprehensive evaluation of the risk of bias in the models. In the open science practices section, none of the studies complied with the disclosure of study protocols (Item 18c), prospective registration (Item 18d), or sharing of analysis code (Item 18f). This may be attributable to a lag in awareness of reporting standards for prediction models in this field and an insufficient consensus on the value of open science practices in clinical prediction modeling. The complete absence of patient and public involvement (Item 19) further disconnected model development from the needs and experiences of end users, potentially underestimating acceptability barriers in clinical deployment. In the Discussion section, none of the studies described strategies for addressing input data quality issues during clinical deployment (Item 27a) or the level of expertise required from users (Item 27b), indicating that current research has not yet fully anticipated the real-world challenges that input data may encounter after model deployment, and that the critical link between evidence generation and clinical deployment lacks necessary preparation.

Taken together, the current evidence exhibits notable deficiencies in both methodological quality and reporting quality, and these deficiencies are heavily concentrated in the Analysis domain and open science practices. This situation underscores a substantial gap between existing postpartum stress urinary incontinence risk prediction models and trustworthy clinical application, and future research must achieve breakthroughs in methodological rigor, transparent reporting, and multicenter external validation.

## 5 Limitations

This study has several limitations. First, this systematic review only included studies published in English and Chinese, and studies reported in other major languages were excluded. Second, most of the included studies adopted retrospective cohort designs for model development and validation, which prevented us from comprehensively assessing the real-world generalizability of the models. Third, all included studies were conducted in Chinese populations, which may limit the extrapolation of the results to Western populations. In addition, with only seven studies, meta-regression lacks sufficient power to reliably detect true moderator effects, and Egger’s test similarly has low statistical power with fewer than 10 studies. Consequently, the non-significant meta-regression and Egger’s test results do not necessarily rule out the possibility of genuine moderator effects or publication bias. Nevertheless, the limitations did not affect the overall evaluation of the models and also, to some extent, reflected the methodological and reporting problems we identified. Finally, most existing models have placed excessive emphasis on improving discriminative performance, as measured by AUC. Due to inconsistent and often incomplete reporting of calibration metrics across included studies, we were unable to perform a pooled analysis of model calibration. However, relying solely on high discriminative ability can be misleading, as it does not guarantee reliable individual risk prediction, which is essential for clinical decision-making. Furthermore, AUC has inherent limitations, such as insufficient sensitivity to changes in absolute risk. Our meta-analysis, which systematically included AUC values and their 95% confidence intervals, helps address these issues by providing a more comprehensive assessment of model performance and accounting for estimation variability.

## 6 Future directions

Future research should focus on the following aspects: (1) conducting large-scale, multicenter prospective cohort studies to provide more robust external validation evidence; (2) strictly adhering to the TRIPOD+AI reporting guidelines and PROBAST+AI methodological assessment standards; (3) routinely reporting model calibration metrics(e.g., calibration curves, calibration intercept, calibration slope and Brier score) and model interpretability metrics, to provide a comprehensive assessment of model performance; (4) incorporating decision curve analysis to evaluate net clinical benefit across a range of threshold probabilities, thereby assessing whether the model would improve clinical decision making; (5) developing standardized measurement protocols, optimizing data fusion strategies, and constructing a more comprehensive model evaluation framework, so as to fully realize the potential of machine learning prediction models in improving clinical predictive reliability and practical utility.

## Data Availability

The original contributions presented in the study are included in the article/Supplementary material, further inquiries can be directed to the corresponding author.
