# Supplementary material for: Machine learning-based prediction model for postpartum stress urinary incontinence risk: a systematic review and meta-analysis
Source: Front Med (Lausanne). 2026 Aug 20;13:1875678. doi: 10.3389/fmed.2026.1875678 (PMC13505298; doi:10.3389/fmed.2026.1875678)
Supplement: Supplementary file 1 [file Image_1.pdf]

Leave−One−Out Sensitivity Analysis(Training Set)

Impact of each study on pooled AUC

Study name	Model	Mean	SE	Variance	Lower	Upper	Z	p	Difference
Zhai et al.,2020	DT	0.939	0.020	0.000	0.887	0.967	21.507	0.000	0.0048
Jiao et al.,2022(1)	DT	0.931	0.023	0.001	0.873	0.963	18.662	0.000	−0.0031
Jiao et al.,2022(2)	LR	0.939	0.020	0.000	0.889	0.968	22.063	0.000	0.0055
Xu et al.,2023(1)	ANN	0.936	0.022	0.000	0.882	0.967	20.217	0.000	0.0025
Xu et al.,2023(2)	DT	0.937	0.021	0.000	0.883	0.967	20.388	0.000	0.0028
Xu et al.,2023(3)	SVM	0.937	0.021	0.000	0.884	0.967	20.555	0.000	0.0032
Xu et al.,2023(4)	LR	0.938	0.021	0.000	0.885	0.967	20.895	0.000	0.0038
Wang et al., 2025(1)	RF	0.927	0.023	0.001	0.870	0.960	18.520	0.000	−0.0070
Wang et al., 2025(2)	LR	0.930	0.023	0.001	0.872	0.963	18.570	0.000	−0.0038
Wang et al., 2025(3)	DT	0.933	0.023	0.001	0.876	0.965	19.081	0.000	−0.0006
Wang et al., 2025(4)	SVM	0.928	0.023	0.001	0.870	0.961	18.542	0.000	−0.0062
Wang et al., 2025(5)	XGB	0.926	0.023	0.001	0.870	0.959	18.663	0.000	−0.0078
Shi et al.,2025(1)	RF	0.933	0.023	0.001	0.876	0.965	19.119	0.000	−0.0004
Shi et al.,2025(2)	DT	0.938	0.021	0.000	0.885	0.967	20.925	0.000	0.0039
Shi et al.,2025(3)	KNN	0.936	0.022	0.000	0.880	0.966	19.800	0.000	0.0017
Shi et al.,2025(4)	SVM	0.935	0.022	0.001	0.878	0.966	19.425	0.000	0.0007
Yang et al.,2025(2)	SVM	0.936	0.022	0.000	0.880	0.966	19.873	0.000	0.0018
Yang et al.,2025(3)	KNN	0.937	0.021	0.000	0.884	0.967	20.566	0.000	0.0033
Yang et al.,2025(4)	XGB	0.921	0.021	0.000	0.870	0.953	19.885	0.000	−0.0127
Yang et al.,2025(5)	DT	0.939	0.020	0.000	0.889	0.967	21.880	0.000	0.0052
Overall		0.934	0.021	0.000	0.881	0.964	20.460	0.000	

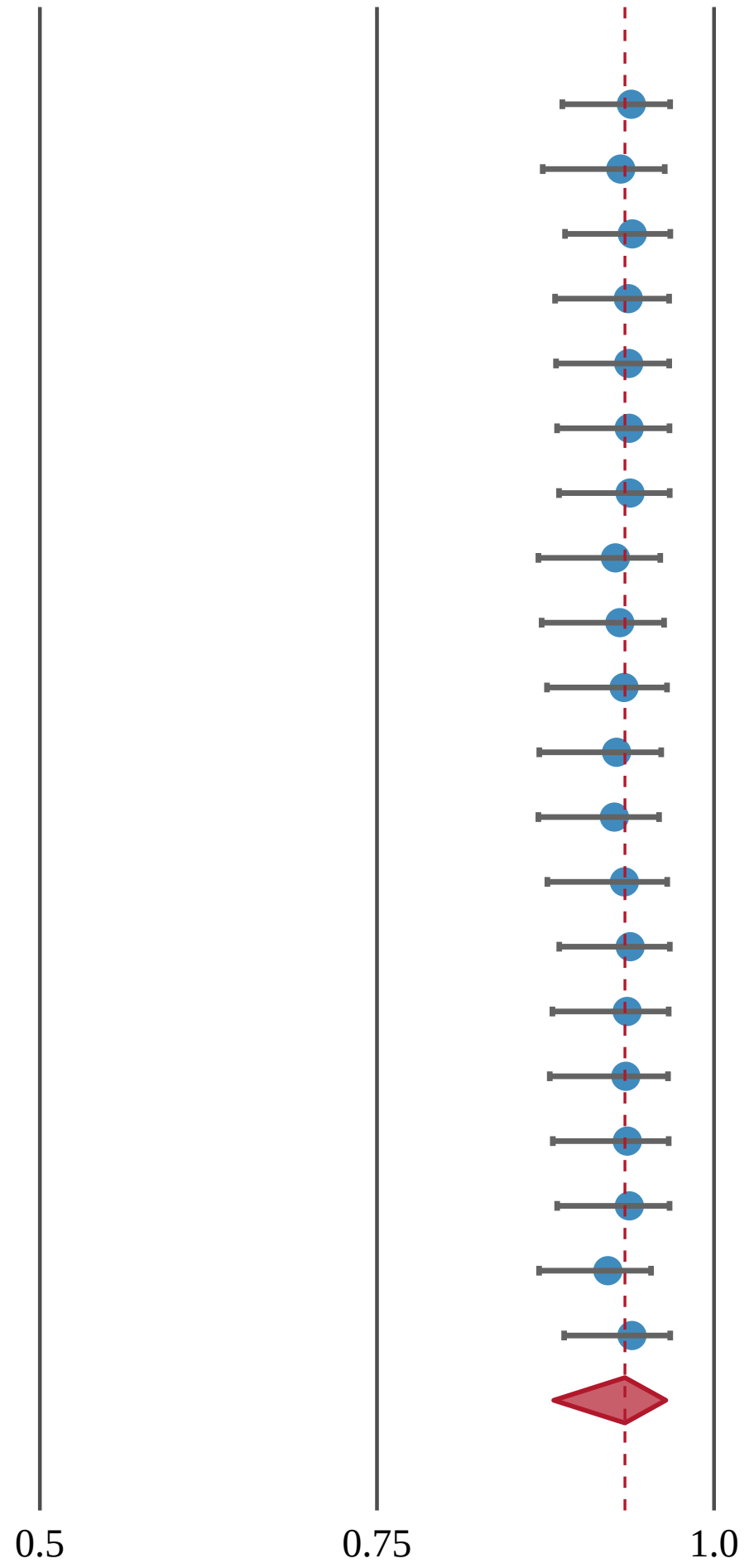

Supplementary Figure S1 The leave-one-out results for 20 training models.

Training Set AUC Meta−Analysis

Robust Variance Estimation

Study name	Model	Mean	SE	Variance	Lower	Upper	Z	p	Total	Weight (%)
Zhai et al.,2020	DT	0.767	0.024	0.001	0.719	0.814	11.017	0.000	490	5.59
Jiao et al.,2022(1)	DT	0.974	0.009	0.000	0.951	0.988	50.218	0.000	360	4.69
Jiao et al.,2022(2)	LR	0.721	0.024	0.001	0.671	0.766	9.119	0.000	360	5.62
Xu et al.,2023(1)	ANN	0.870	0.035	0.001	0.786	0.925	10.435	0.000	115	4.96
Xu et al.,2023(2)	DT	0.856	0.037	0.001	0.809	0.955	9.558	0.000	115	4.49
Xu et al.,2023(3)	SVM	0.843	0.042	0.002	0.738	0.904	8.100	0.000	115	4.97
Xu et al.,2023(4)	LR	0.815	0.044	0.002	0.705	0.879	7.097	0.000	115	5.08
Wang et al., 2025(1)	RF	0.990	0.002	0.000	0.985	0.994	213.422	0.000	966	5.27
Wang et al., 2025(2)	LR	0.978	0.006	0.000	0.965	0.989	78.073	0.000	966	5.00
Wang et al., 2025(3)	DT	0.948	0.014	0.000	0.917	0.973	31.360	0.000	966	5.00
Wang et al., 2025(4)	SVM	0.989	0.004	0.000	0.980	0.996	119.805	0.000	966	4.47
Wang et al., 2025(5)	XGB	0.993	0.003	0.000	0.988	0.998	193.256	0.000	966	4.25
Shi et al.,2025(1)	RF	0.944	0.010	0.000	0.924	0.964	43.512	0.000	966	5.39
Shi et al.,2025(2)	DT	0.815	0.019	0.000	0.778	0.853	16.464	0.000	428	5.61
Shi et al.,2025(3)	KNN	0.900	0.015	0.000	0.870	0.929	26.576	0.000	428	5.49
Shi et al.,2025(4)	SVM	0.925	0.012	0.000	0.901	0.949	34.708	0.000	428	5.46
Yang et al.,2025(2)	SVM	0.896	0.027	0.001	0.844	0.949	14.784	0.000	361	4.94
Yang et al.,2025(3)	KNN	0.843	0.026	0.001	0.792	0.894	13.182	0.000	361	5.39
Yang et al.,2025(4)	XGB	0.999	0.000	0.000	0.998	1.000	1029.516	0.000	361	2.90
Yang et al.,2025(5)	DT	0.734	0.036	0.001	0.663	0.804	6.506	0.000	361	5.44
Overall		0.917	0.046	0.002	0.790	0.970	9.060	0.000		100

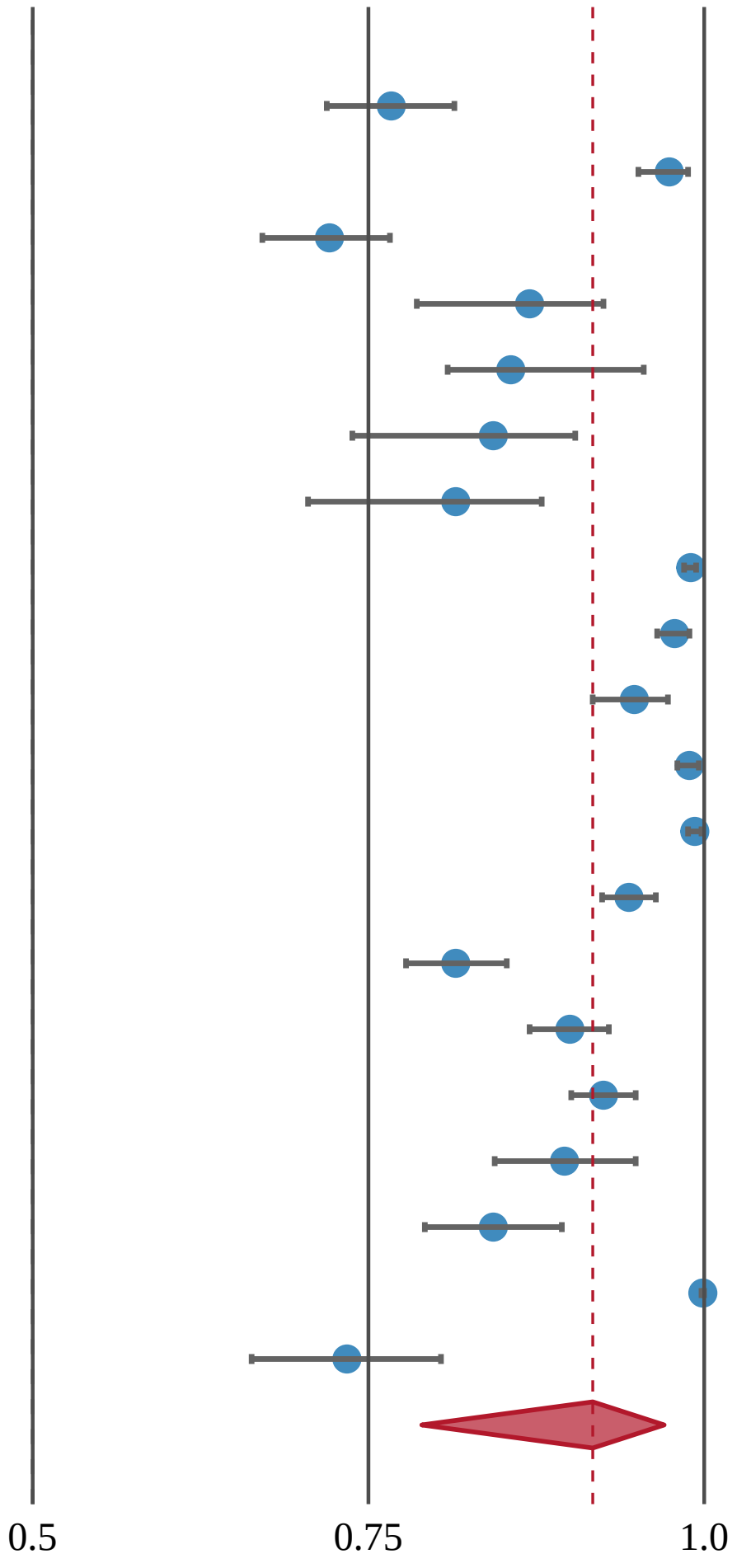

Supplementary Figure S2 Forest plot of the pooled AUC estimates for 20 training models based on robust variance estimation.

Leave−One−Out Sensitivity Analysis (Validation Set)

Impact of each study on pooled AUC

Study name	Model	Mean	SE	Variance	Lower	Upper	Z	p	Difference
Zhai et al.,2020	DT	0.897	0.024	0.001	0.842	0.935	16.751	0.000	0.0071
Jiao et al.,2022(1)	DT	0.892	0.026	0.001	0.831	0.933	15.147	0.000	0.0020
Jiao et al.,2022(2)	LR	0.896	0.025	0.001	0.838	0.934	16.144	0.000	0.0056
Xu et al.,2023(1)	ANN	0.892	0.026	0.001	0.831	0.932	15.091	0.000	0.0013
Xu et al.,2023(2)	DT	0.894	0.025	0.001	0.835	0.934	15.705	0.000	0.0039
Xu et al.,2023(3)	SVM	0.894	0.025	0.001	0.835	0.933	15.611	0.000	0.0036
Xu et al.,2023(4)	LR	0.891	0.026	0.001	0.830	0.932	14.979	0.000	0.0007
Wang et al.,2025(1)	RF	0.877	0.025	0.001	0.820	0.918	15.001	0.000	−0.0127
Wang et al.,2025(2)	LR	0.883	0.027	0.001	0.821	0.925	14.422	0.000	−0.0077
Wang et al.,2025(3)	DT	0.887	0.027	0.001	0.824	0.929	14.472	0.000	−0.0034
Wang et al.,2025(4)	SVM	0.880	0.026	0.001	0.820	0.921	14.661	0.000	−0.0106
Wang et al.,2025(5)	XGB	0.878	0.025	0.001	0.820	0.919	14.921	0.000	−0.0125
Shi et al.,2025(1)	RF	0.890	0.026	0.001	0.828	0.931	14.730	0.000	−0.0006
Shi et al.,2025(2)	DT	0.895	0.025	0.001	0.836	0.934	15.744	0.000	0.0044
Shi et al.,2025(3)	KNN	0.891	0.026	0.001	0.829	0.932	14.891	0.000	0.0005
Shi et al.,2025(4)	SVM	0.891	0.026	0.001	0.829	0.932	14.910	0.000	0.0007
Yang et al.,2025(1)	RF	0.892	0.026	0.001	0.832	0.933	15.188	0.000	0.0020
Yang et al.,2025(2)	SVM	0.894	0.025	0.001	0.834	0.933	15.534	0.000	0.0034
Yang et al.,2025(3)	KNN	0.893	0.025	0.001	0.834	0.933	15.479	0.000	0.0032
Yang et al.,2025(4)	XGB	0.894	0.025	0.001	0.835	0.934	15.585	0.000	0.0037
Yang et al.,2025(5)	DT	0.895	0.025	0.001	0.838	0.934	16.038	0.000	0.0052
Wang et al.,2025(1)	SVM	0.889	0.026	0.001	0.827	0.930	14.774	0.000	−0.0013
Wang et al.,2025(2)	LR	0.891	0.026	0.001	0.830	0.932	14.974	0.000	0.0007
Wang et al.,2025(3)	RF	0.890	0.026	0.001	0.828	0.931	14.848	0.000	−0.0003
Wang et al.,2025(4)	XGB	0.889	0.026	0.001	0.827	0.930	14.779	0.000	−0.0016
Overall		0.890	0.025	0.001	0.832	0.930	15.512	0.000	

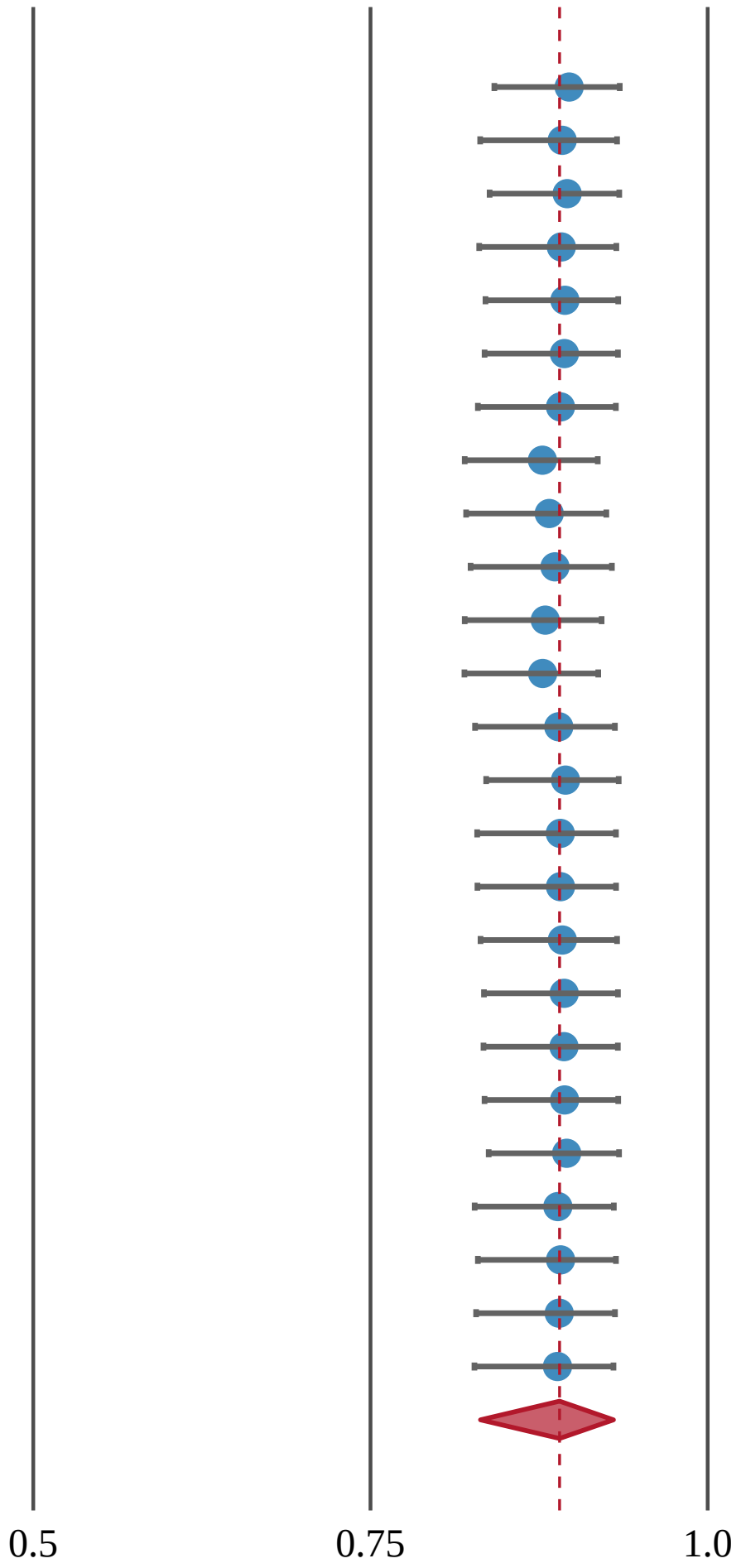

Supplementary Figure S3 The leave-one-out results for 25 validation models.

Validation Set AUC Meta−Analysis

Robust Variance Estimation

Study name	Model	Mean	SE	Variance	Lower	Upper	Z	p	Total	Weight (%)
Zhai et al.,2020	DT	0.602	0.045	0.002	0.513	0.691	2.258	0.024	217	4.43
Jiao et al.,2022(1)	DT	0.842	0.032	0.001	0.772	0.897	10.725	0.000	145	4.35
Jiao et al.,2022(2)	LR	0.689	0.040	0.002	0.607	0.764	4.719	0.000	145	4.44
Xu et al.,2023(1)	ANN	0.857	0.061	0.004	0.699	0.938	5.855	0.000	48	3.79
Xu et al.,2023(2)	DT	0.746	0.090	0.008	0.533	0.885	2.740	0.006	48	3.77
Xu et al.,2023(3)	SVM	0.763	0.087	0.008	0.561	0.901	3.032	0.002	48	3.73
Xu et al.,2023(4)	LR	0.876	0.064	0.004	0.682	0.933	5.872	0.000	48	3.79
Wang et al.,2025(1)	RF	0.995	0.002	0.000	0.990	0.999	215.600	0.000	242	3.48
Wang et al.,2025(2)	LR	0.978	0.006	0.000	0.965	0.989	78.073	0.000	242	4.23
Wang et al.,2025(3)	DT	0.948	0.014	0.000	0.917	0.973	31.360	0.000	242	4.23
Wang et al.,2025(4)	SVM	0.989	0.004	0.000	0.980	0.996	119.805	0.000	242	3.96
Wang et al.,2025(5)	XGB	0.993	0.003	0.000	0.988	0.998	193.256	0.000	242	3.84
Shi et al.,2025(1)	RF	0.906	0.022	0.000	0.863	0.948	18.724	0.000	183	4.29
Shi et al.,2025(2)	DT	0.750	0.033	0.001	0.685	0.815	7.538	0.000	183	4.45
Shi et al.,2025(3)	KNN	0.881	0.024	0.001	0.834	0.929	15.721	0.000	183	4.35
Shi et al.,2025(4)	SVM	0.878	0.024	0.001	0.830	0.926	15.435	0.000	183	4.35
Yang et al.,2025(1)	RF	0.837	0.042	0.002	0.755	0.919	8.055	0.000	155	4.16
Yang et al.,2025(2)	SVM	0.779	0.065	0.004	0.652	0.905	4.323	0.000	155	3.96
Yang et al.,2025(3)	KNN	0.789	0.057	0.003	0.677	0.901	5.058	0.000	155	4.06
Yang et al.,2025(4)	XGB	0.772	0.055	0.003	0.665	0.879	4.982	0.000	155	4.17
Yang et al.,2025(5)	DT	0.701	0.055	0.003	0.593	0.808	3.665	0.000	155	4.29
Wang et al.,2025(1)	SVM	0.924	0.033	0.001	0.860	0.988	12.960	0.000	109	3.26
Wang et al.,2025(2)	LR	0.875	0.041	0.002	0.795	0.955	9.145	0.000	109	3.90
Wang et al.,2025(3)	RF	0.900	0.037	0.001	0.827	0.973	10.774	0.000	109	3.69
Wang et al.,2025(4)	XGB	0.931	0.031	0.001	0.870	0.992	13.784	0.000	109	3.01
Overall		0.825	0.074	0.005	0.637	0.927	4.389	0.000		100

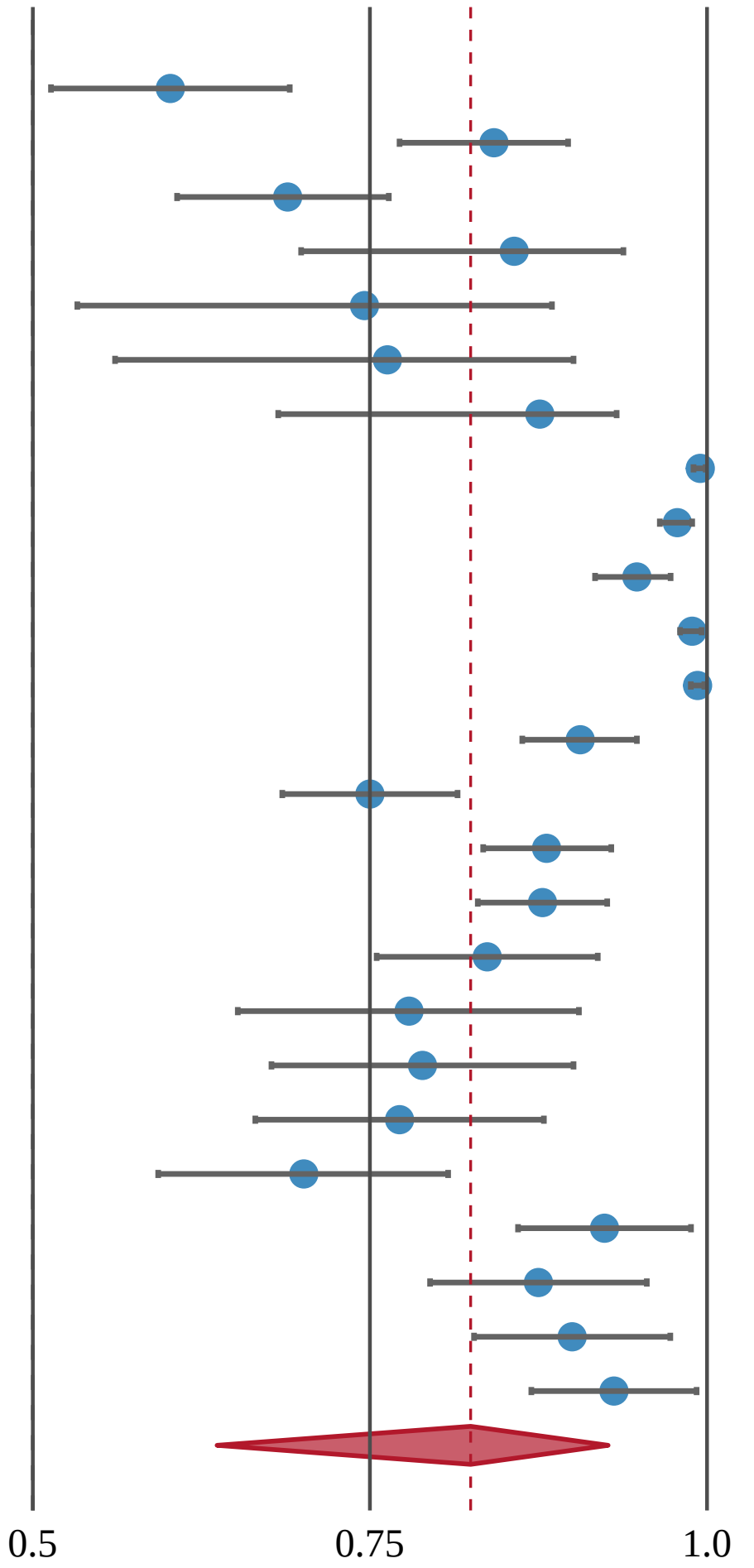

Supplementary Figure S4 Forest plot of the pooled AUC estimates for 25 validation models based on robust variance estimation.
