# Supplementary material for: Machine learning-based prediction model for postpartum stress urinary incontinence risk: a systematic review and meta-analysis
Source: Front Med (Lausanne). 2026 Aug 20;13:1875678. doi: 10.3389/fmed.2026.1875678 (PMC13505298; doi:10.3389/fmed.2026.1875678)
Supplement: Supplementary file 3 [file Table_1.docx]

**Supplementary Table S1 Dataset-Model Stratification & Interaction Effects**

| **Factor** | **Variance** | **σ** | **ρ** |
| --- | --- | --- | --- |
| **A: Training model** | | | |
| **Dataset** | 0.464 | 0.681 | / |
| **Dataset/Model** | 1.397 | 1.182 | 0.751 |
| **B: Validation model** | | | |
| **Dataset** | 1.259 | 1.122 | / |
| **Dataset/Model** | 0.213 | 0.461 | 0.144 |

**Note: σ:** standard deviation；**ρ:** Correlation coefficient between effect sizes.

**Supplementary Table S2 Meta-regression results**

|  | **β** | | | **SE** | | **95%CI** | ***P*** | **I²** | |
| --- | --- | --- | --- | --- | --- | --- | --- | --- | --- |
| **A: Training model** | | | | | | | | | |
| **Sample size** | 0.002 | | | 0.001 | | [0.001, 0.004] | **0.010** | 97.2% | |
| **Public year** | 0.397 | | | 0.212 | | [-0.049, 0.842] | 0.078 | 97.9% | |
| **B: Validation model** | | | | | | | | | |
| **Sample size** | | 0.010 | 0.004 | | [0.003, 0.018] | | **0.008** | | 91.7% |
| **Public year** | | 0.410 | 0.169 | | [0.060, 0.760] | | **0.024** | | 91.8% |

**Note:** Meta-regression utilizes the Knapp-Hartung adjustment method and maximum likelihood estimation. **β:** Regression coefficient; **95%CI**: 95% confidence interval; *P* **value** < 0.05 indicates that the effect of the moderator variable on the pooled AUC is statistically significant and represents a significant source of heterogeneity in AUC estimates across different predictive models.

**Supplementary Table S3 Leave‑one‑out analysis results**

| **Analysis level** | **Most influential model/study** | **Original I²** | **I² after exclusion** |
| --- | --- | --- | --- |
| **A: Training model** | | | |
| **By model** | Yang et al.,2025(3) | 98.2% | 97.6% |
| **By study** | Wang et al.,2025 (5 models) | 98.2% | 97.1% |
| **B: Validation model** | | | |
| **By model** | Wang L et al.,2025(1) | 93.6% | 92.2% |
| **By study** | Wang L et al.,2025 (5 models) | 93.6% | 72.6% |

**Note:** **Original I^2^**: original heterogeneity I^2^ value; **I^2^ after exclusion**: heterogeneity I^2^ value after excluding the corresponding model/study.

**Supplementary Table S4 Egger's test results**

|  | **β₀** | **95%CI** | ***P*** | ***t*** |
| --- | --- | --- | --- | --- |
| **Training model** | 0.203 | [-1.628, 3.415] | 0.370 | 0.627 |
| **Validation model** | 0.325 | [-1.483, 2.133] | 0.640 | 0.508 |

**Note:** All publication bias tests were performed using robust variance estimation (RVE). **β₀:** intercept of the regression line; *P* **value** < 0.05 suggests publication bias.

**Supplementary Table S5 Subgroup analysis results of training model**

|  | **Model number** | **Mean** | **SE** | **Variance** | **95%CI** | ***P*** | **I²** |
| --- | --- | --- | --- | --- | --- | --- | --- |
| **Types of included predictors (Total between: Q value = 5.069, *P* = 0.079)** | | | | | | | |
| Clinical features + PFU + PFEMG | 12 | 0.892 | 0.031 | 0.001 | [0.818, 0.938] | <0.001 | 92.8% |
| Clinical features | 6 | 0.970 | 0.026 | 0.001 | [0.889, 0.992] | <0.001 | 98.4% |
| Clinical features + PFEMG | 2 | 0.906 | 0.255 | 0.065 | [0.000, 1.000] | 0.112 | 97.9% |
| **Sample size (Total between: Q value = 2.231, *P* = 0.128)** | | | | | | | |
| ＞394 | 10 | 0.952 | 0.022 | 0.000 | [0.892, 0.979] | <0.001 | 98.1% |
| ≤394 | 10 | 0.892 | 0.049 | 0.002 | [0.762, 0.955] | <0.001 | 94.6% |
| **Model types (Total between:** **Q value = 12.922, *P* = 0.044)** | | | | | | | |
| DT | 6 | 0.873 | 0.061 | 0.004 | [0.712, 0.950] | <0.001 | 93.2% |
| SVM | 4 | 0.935 | 0.073 | 0.005 | [0.702, 0.989] | <0.001 | 95.2% |
| LR | 3 | 0.886 | 0.212 | 0.045 | [0.167, 0.997] | 0.068 | 98.3% |
| RF | 2 | 0.976 | 0.255 | 0.065 | [0.001, 1.000] | 0.062 | 96.9% |
| XGB | 2 | 0.996 | 0.255 | 0.065 | [0.000, 1.000] | 0.052 | 90.1% |
| KNN | 2 | 0.876 | 0.200 | 0.040 | [0.210, 0.995] | 0.061 | 73.6% |
| ANN | 1 | 0.870 | / | / | [0.786, 0.925] | / | / |

**Note:** All subgroup analyses were performed using robust variance estimation (RVE). **Total between:** Compare whether there are differences between subgroup; **PFEMG:** Pelvic Floor Electromyography; **PFU:** Pelvic Floor Ultrasound; **DT:** Decision Tree; **SVM:** Support Vector Machine; **LR:** Logistic Regression; **RF:** Random Forest; **XGB:** Extreme Gradient Boosting; **KNN:** K-Nearest Neighbors; **ANN:** Artificial Neural Network; *P* value < 0.05 indicates differences among subgroups.

**Supplementary table S6 Subgroup analysis results of validation model**

|  | **Model number** | **Mean** | **SE** | **Variance** | **95%CI** | ***P*** | **I²** |
| --- | --- | --- | --- | --- | --- | --- | --- |
| **Types of included predictors (Total between:** **Q value = 18.240, *P* < 0.001)** | | | | | | | |
| Clinical features + PFU + PFEMG | 13 | 0.821 | 0.023 | 0.001 | [0.771, 0.862] | <0.001 | 50.3% |
| Clinical features | 6 | 0.974 | 0.038 | 0.001 | [0.846, 0.996] | <0.001 | 95.2% |
| Clinical features + PFU | 4 | 0.900 | 0.026 | 0.001 | [0.838, 0.941] | <0.001 | 0% |
| Clinical features + PFEMG | 2 | 0.772 | 0.252 | 0.063 | [0.013, 0.999] | 0.279 | 87.8% |
| **Sample size (Total between: Q value = 2.334, *P* = 0.126)** | | | | | | | |
| ＞155 | 15 | 0.910 | 0.037 | 0.001 | [0.815, 0.959] | <0.001 | 94.9% |
| ≤155 | 10 | 0.839 | 0.032 | 0.001 | [0.768, 0.891] | <0.001 | 43.7% |
| **Model types (Total between:** **Q value = 4.977, *P* = 0.547)** | | | | | | | |
| DT | 6 | 0.790 | 0.078 | 0.006 | [0.600, 0.905] | <0.001 | 88.6% |
| SVM | 5 | 0.907 | 0.088 | 0.008 | [0.638, 0.982] | <0.001 | 88.5% |
| LR | 4 | 0.864 | 0.175 | 0.030 | [0.305, 0.989] | 0.037 | 91.4% |
| RF | 4 | 0.941 | 0.107 | 0.011 | [0.576, 0.995] | <0.001 | 92.2% |
| XGB | 3 | 0.949 | 0.222 | 0.049 | [0.128, 1.000] | 0.043 | 92.2% |
| KNN | 2 | 0.848 | 0.236 | 0.056 | [0.072, 0.998] | 0.140 | 57.1% |
| ANN | 1 | 0.857 | / | / | [0.699, 0.938] | / | / |

**Note:** All subgroup analyses were performed using robust variance estimation (RVE). **Total between:** Compare whether there are differences between subgroup; **PFEMG:** Pelvic Floor Electromyography; **PFU:** Pelvic Floor Ultrasound; **DT:** Decision Tree; **SVM:** Support Vector Machine; **LR:** Logistic Regression; **RF:** Random Forest; **XGB:** eXtreme Gradient Boosting; **KNN:** K-Nearest Neighbors; **ANN:** Artificial Neural Network; *P* value < 0.05 indicates differences among subgroups.
